# Contribution of PDGFRα-positive cells in maintenance and injury responses in mouse large vessels

**DOI:** 10.1038/s41598-021-88126-6

**Published:** 2021-04-21

**Authors:** Kenichi Kimura, Karina Ramirez, Tram Anh Vu Nguyen, Yoshito Yamashiro, Aiko Sada, Hiromi Yanagisawa

**Affiliations:** 1 Life Science Center for Survival Dynamics, Tsukuba Advanced Research Alliance (TARA), University of Tsukuba, 1-1-1 Tennodai, Tsukuba, Ibaraki 305-8577 Japan; 2 Ph.D. Program in Human Biology, School of Integrative and Global Majors, University of Tsukuba, Tsukuba, Japan; 3 International Research Center for Medical Sciences (IRCMS), Kumamoto University, Kumamoto, Japan; 4 Faculty of Medicine, University of Tsukuba, Tsukuba, Japan

**Keywords:** Cardiovascular biology, Adult stem cells

## Abstract

The maladaptive remodeling of vessel walls with neointima formation is a common feature of proliferative vascular diseases. It has been proposed that neointima formation is caused by the dedifferentiation of mature smooth muscle cells (SMCs). Recent evidence suggests that adventitial cells also participate in neointima formation; however, their cellular dynamics are not fully understood. In this study, we utilized a lineage tracing model of platelet-derived growth factor receptor alpha (PDGFRa) cells and examined cellular behavior during homeostasis and injury response. PDGFRa marked adventitial cells that were largely positive for Sca1 and a portion of medial SMCs, and both cell types were maintained for 2 years. Upon carotid artery ligation, PDGFRa-positive (+) cells were slowly recruited to the neointima and exhibited an immature SMC phenotype. In contrast, in a more severe wire denudation injury, PDGFRa+ cells were recruited to the neointima within 14 days and fully differentiated into SMCs. Under pressure overload induced by transverse aortic constriction, PDGFRa+ cells developed marked adventitial fibrosis**.** Taken together**,** our observations suggest that PDGFRa+ cells serve as a reservoir of adventitial cells and a subset of medial SMCs and underscore their context-dependent response to vascular injuries.

## Introduction

The increased proliferation of smooth muscle cells (SMCs) and pathological remodeling of the vessel walls are associated with a variety of vascular diseases such as atherosclerosis, in-stent restenosis, and vein grafts, and account for major complications that can involve morbidity and mortality^1–3^. Traditionally, it is believed that adult blood vessels contain terminally differentiated SMCs and that an injury induces the de-differentiation of mature SMCs through injury-evoked mechanical stimuli, the activation of endothelial cells, and the secretion of cytokines. These stimuli convert mature SMCs into an immature synthetic phenotype, resulting in increased proliferation and migration as well as the secretion of extracellular matrix (ECM) through a phenomenon known as phenotypic modulation^4,5^.

Recent advances in lineage tracing allow us to chase the fate of SMCs upon vascular injury. In lineage tracing, cells are genetically labeled and traced for a certain period by relying on a tamoxifen-inducible Cre recombinase (CreER) driven by the promoter/enhancer of a gene of interest. Upon tamoxifen injection, Cre enters the nucleus and activate the expression of Cre reporters, such as Rosa-tdTomato, Rosa-lacZ, and Rosa-eYFP. Since Cre recombination is irreversible and inheritable, the observable label is passed on to the progeny of the Cre-expressing cells^6^.

The promoters of myosin heavy chain (Myh11) and transgelin (SM22α)— both markers of differentiated SMCs—have been used for the lineage tracing of SMCs in mechanical injury models such as carotid artery ligation and denudation injury, and in a murine atherosclerosis model^7,8^. SMC-derived cells also convert their phenotype to macrophages, mesenchymal stem cells, or a subpopulation of resident progenitor cells in the adventitia^9–11^.

Recently, adventitia-derived cells have gained attention as a source of neointima cells^12^. Adventitia is composed of fibroblasts, macrophages, inflammatory cells, and vasa vasorum in the case of humans, and is also suggested to contain vascular progenitor cells marked with Sca1^13,14^. Some studies have provided clues regarding the dynamism of blood vessels, arguing that stem/progenitor cells residing in vessel walls are responsible for the proliferative state of adult vessels and potentially serve essential roles in vascular diseases^15,16^. Progenitor cells that reside in the adventitia have been characterized by their ability to proliferate and differentiate into different cell lineages, which are usually positive for markers such as Sca1, Gli, CD34, and CD45.

However, the level of adventitia-derived cell participation during various vascular remodeling events has not been thoroughly elucidated. This is partly due to the paucity of Cre mice that can induce recombination in the adventitia. A lineage tracing study using Gli1-CreER showed that Gli1+ cells express Sca1, CD34, and platelet-derived growth factor receptor β (PDGFRb) and possess mesenchymal stem cell (MSC)-like characteristics. These cells differentiate into SMCs in the neointima after wire injury of the femoral artery (i.e., acute injury repair) and under hyperlipidemic and chronic kidney disease conditions (i.e., chronic injury repair) and can also de-differentiate into osteoblastic cells^17^. Another study using Sca1-CreER has shown that Sca1+ adventitial cells, which overlap approximately 70% with platelet-derived growth factor receptor α (PDGFRa) but only 20% with PDGFRb contribute SMCs after a severe injury such as arterial anastomosis and exhibit a high proliferative capacity^18^. Surprisingly, these Sca1+ cells were not observed in the neointima induced by wire injury.

While several Pdgfra-CreER mouse lines have been established, relatively few studies on the lineage tracing of adventitial cells have focused on the vascular wall^16,19^. In the present study, we characterized a Pdgfra-CreER knock-in line and examined PDGFRa-derived cells during homeostasis and injury response in large vessels to investigate the complex nature of vascular remodeling and the contribution of PDGFRa+ cells in vivo. We utilized three types of mechanical insults for injury models: carotid artery ligation, carotid artery wire injury, and transverse aortic constriction. Our data provide novel evidence that demonstrates the level of contribution of PDGFRa+ adventitial cell and medial SMC contribution to vascular remodeling in murine injury models.

## Results

### Characterization of PDGFRa+ cells in vascular walls

**Figure 1:**
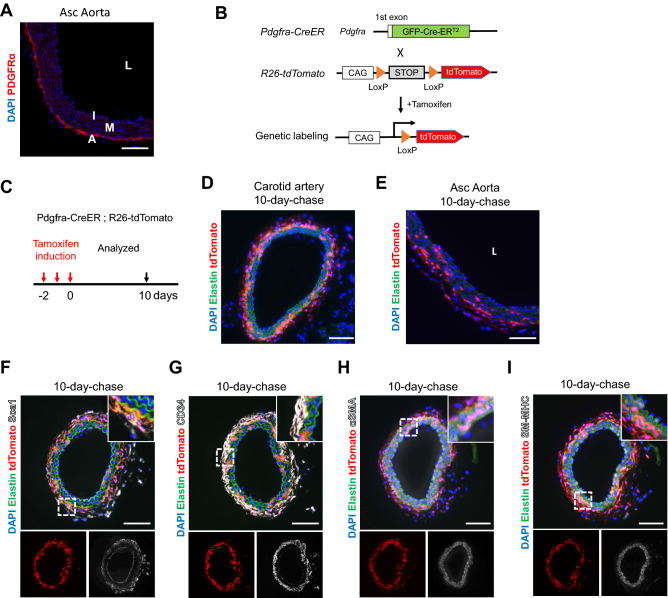
Genetic labeling of PDGFRa+ cells in the carotid artery. (**A**) Immunostaining for PDGFRa in the mouse ascending aorta at 2 months of age. Scale bar: 100 µm. (**B**) Schematic showing genetic lineage tracing via Pdgfra-CreER*.* To express the fusion protein instead of endogenous PDGFRα, the cDNA of green fluorescent protein (GFP), Cre recombinase (Cre), and mutated estrogen receptor ligand-binding domain (ER^T2^) was inserted into the translational start site of the PDGFRα locus. (**C**) Schematic outlining the experimental strategy. (**D**, **E**) Representative images of the initial labeling of tdTomato+ cells in the carotid artery (**D**) and ascending aorta (**E**) at 10-day-chase after tamoxifen administration. Asc Aorta: ascending aorta. Scale bars: 100 µm. (**F**–**I**) Immunostaining for Sca1 (**F**), CD34 (**G**), αSMA (**H**), and SM-MHC (**I**) on carotid artery sections from 10-days-chase. The dotted box area is shown in the inset with higher magnification. Scale bars: 100 µm. See also Figures S1 and S2.

To investigate the contribution of PDGFRa+ adventitial cells in vascular injury response, we first examined PDGFRa expression in the adult aorta by immunostaining (Fig. 1A). While PDGFRa was strongly expressed in the adventitia, no expression was detected in the medial or intima layer, thus confirming its suitability as an adventitial marker. We used Pdgfra-CreER mice, in which green fluorescent protein (GFP) was fused with CreER and knocked into the first ATG of the *Pdgfra* locus (Fig. 1B); thus, the expression was driven by its own promoter^20^. Although > 95% of GFP-positive cells have been reported to be co-expressed with tdTomato cells in vitro^20^, GFP was not detected in the adult aorta or carotid arteries (Figure S1A).

To verify inducible Cre expression in the adventitia in vivo, we crossed Pdgfra-CreER driver mice with R26-tdTomato mice for genetic labeling (Fig. 1B). Mice were treated with tamoxifen (100 ug/g body weight) or a vehicle at 2 months of age, and leakiness in the construct was tested 10 days after the final injection. No tdTomato expression was detected in the aortic wall without tamoxifen (Figure S1A). We then treated Pdgfra-CreER; R26 tdTomato mice with tamoxifen and harvested the carotid artery and ascending aorta on day 10 to examine the initial labeling (Figs. 1C–E). While labeled cells largely resided within the adventitia, some cells were also detected in the medial layers of both vessels at day 10.

To examine the detailed cell types of PDGFRa+ cells in the vessel walls, we performed immunostainings against cell lineage markers. The staining of carotid arteries for progenitor cell markers showed that tdTomato+ cells in the adventitial layer largely overlapped with Sca1+ and CD34+ at 10 days after tamoxifen administration (Figs. 1F, G). TdTomato+ cells with high and low Sca1 expression were observed in the adventitia. Additionally, we observed that tdTomato+ cells in the media expressed conventional SMC markers, alpha-smooth muscle actin (αSMA), and smooth muscle-myosin heavy chain (SM-MHC) (Figs. 1H, I, respectively) but not endothelial cell marker CD31 (Figure S1B). Similarly, we detected tdTomato+/Sca1+and tdTomato+/CD34+cells in the adventitia (Figures S2A, B) and tdTomato+/αSMA+ and tdTomato+/SM-MHC+ SMCs in the medial layers of the ascending aorta (Figures S2C, D), but not CD31 at 10-day-chase (Figure S2E). These observations suggest that PDGFRa labeled two distinct cell populations—one being adventitial cells that largely co-expressed progenitor markers, while the other is a subset of differentiated SMCs. It is less likely that PDGFRa+ adventitial cells differentiated into SMCs within a short period of time.

**Figure 2:**
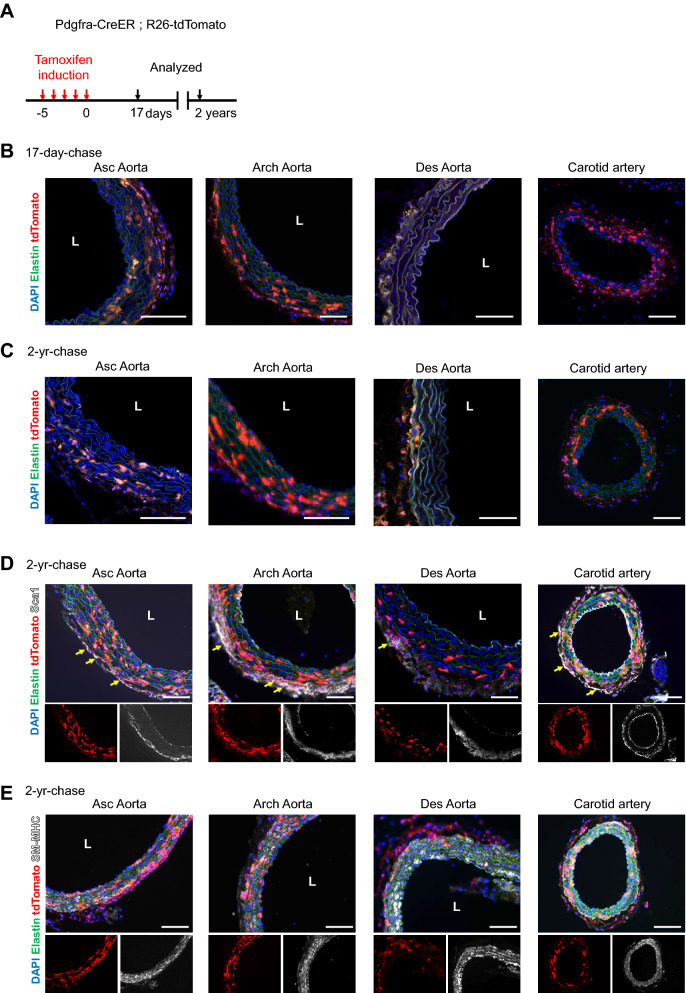
The fate of of PDGFRa+ cells during a long-term homeostasis of the aortic wall. (**A**) Schematic outlining the experimental strategy. (**B**) Representative images of the initial labeling at 2 months of age in different portions of large arteries. L: lumen, Asc Aorta: ascending aorta, Des Aorta: descending aorta. Scale bars: 100 µm. (**C**) Representative images of 2-year-chase in different portions of larger arteries. L: lumen, Asc Aorta: ascending aorta, Des Aorta: descending aorta. Scale bars: 100 µm. (**D**, **E**) Immunostaining for Sca1 (**D**) and SM-MHC (**E**) in the aortas at 2-year-chase. Arrows indicate tdTomato+ /Sca1+ adventitial cells. L: lumen, Asc Aorta: ascending aorta, Des Aorta: descending aorta. Scale bars: 100 µm. See also Figure S3.

The turnover of SMCs in the large arteries was previously studied, and their half-life was determined to be approximately 270 ~ 400 days^21^. Another study also assessed SMC turnover in developing and adult femoral arteries in the context of injury response using the fate map of NG2+ and CD146+ immature SMCs^22^. In the present study, we sought to investigate the long-term survival of PDGFRa-labeled cells and their progeny. For this experiment, we injected tamoxifen over 5 days instead of 3 days to increase the labeling efficiency and maintained animals for 2 years in a standard housing condition (Fig. 2A). The initial labeling of 5-day tamoxifen injection was examined at 17 days after administration (Fig. 2B), and tdTomato+ cells were compared with a 2-year-chase in the aorta from ascending to descending as well as carotid arteries (Fig. 2C). At a 2-year-chase, Sca1 immunostaining showed Sca1+/tdTomato+ cells in the adventitia of ascending, arch, carotid arteries—even after 2 years of lineage tracing (see arrows, Fig. 2D). In contrast, the descending aorta contained much fewer tdTomato+ cells in the adventitia (see Des Aorta in Fig. 2D), which was already observed at the initial labeling (Fig. 2B). The medial layers of ascending, arch, and carotid arteries all exhibited a high number of tdTomato+ cells that co-expressed SM-MHC (Fig. 2E). However, in the descending aorta; tdTomato+ medial cells were much less commonly detected (Fig. 2E, Des Aorta). Staining for CD31 validated that labeled PDGFRa+ cells did not contribute to endothelial cells in any of the aortas (Figure S3). Taken together, the number of PDGFRa+ cells is maintained in the medial layers (even after 2 years) and are involved in the homeostasis of adventitial cells and SMCs in vessel walls. Additionally, the descending aorta—which is derived from the lateral mesoderm and has a distinct embryonic origin from the rest of the large arteries included in this study (i.e. neural crest)—exhibited less efficient labeling, which demonstrates the heterogeneity of PDGFRa-expressing cells among thoracic aortas.

### PDGFRa+ cells contribute to neointima formation

Neointimal hyperplasia is a common result of atherosclerosis and atherosclerotic occlusion treatments (e.g., angioplasty) and has been extensively studied using different types of injury models^23^. In this study, we employed two types of neointima in the carotid artery: (a) a complete ligation that induces neointima due to flow cessation and resultant changes in flow shear stress; (b) wire injury in which the endothelial cell layer is denudated.

**Figure 3:**
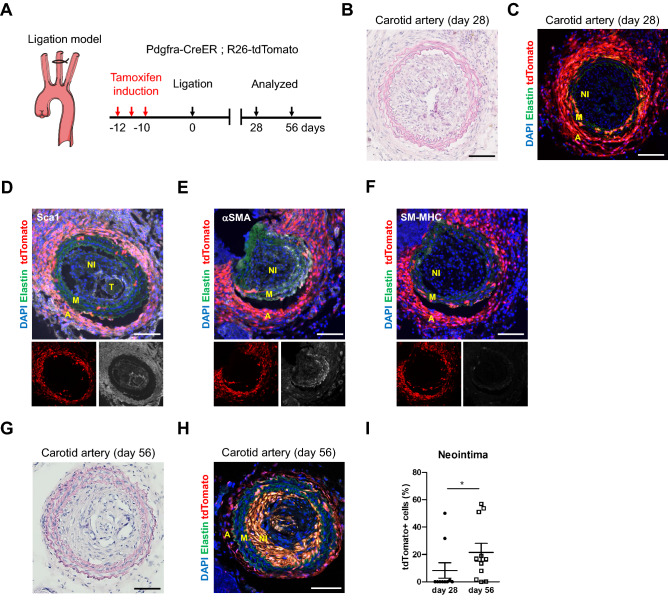
Contribution of PDGFRa+ cells to neointima formation following carotid artery ligation. (**A**) Schematic outlining the experimental strategy. (**B**) Hematoxylin eosin-stained carotid artery at 28 days after ligation. Scale bar: 100 µm. (**C**) Fluorescence image showing the distribution of tdTomato+ cells at 28 days after ligation. NI: neointima, M: media, A: adventitia. Scale bar: 100 µm. (**D**–**F**) Immunostaining for Sca1 (**D**), αSMA (**E**), and SM-MHC (**F**) on carotid artery sections at 28 days after ligation. NI: neointima, M: media, A: adventitia, T: thrombus. Scale bars: 100 µm. (**G**) Hematoxylin eosin-stained carotid artery at 56 days after ligation. Scale bar: 100 µm. (**H**) Fluorescence image showing the contribution of tdTomato+ cells to neointima at 56 days after ligation. NI: neointima, M: media, A: adventitia. Scale bar: 100 µm. (**I**) Quantification of the contribution of tdTomato+ cells to neointima formation. Data are presented as the mean ± SEM, 28 days, *N* = 10, 56 days, *N* = 11, **p < 0.01, chi-square test. See also Figure S4.

To determine whether PDGFRa-derived tdTomato+ cells contribute to neointima formation after carotid artery ligation, we injected tamoxifen at 2 months of age and performed ligation after 10 days (Fig. 3A). Tissues were analyzed at 28 days after ligation when a neointima was established and at 56 days when the long-term effects of cell contributions could be evaluated. Hematoxylin eosin staining displayed neointima formation after ligation (Fig. 3B). The sham-operated control showed no neointima and 91.8% of Sca1+ cells in the adventitia were tdTomato+ cells (n = 3, Figures S4A–C). Serial sections showed that tdTomato+ cells in the adventitia and media increased, whereas tdTomato+ cells were not detected in the neointima at 28 days after ligation, with a few exceptions (e.g., NI in Fig. 3C). Immunostaining for Sca1 revealed that a large number of adventitial tdTomato+ cells were Sca1+ and that Sca1 expression was also detected on endothelial cells in the neointima (Fig. 3D). αSMA—a marker for immature SMC—was positive in the medial layer and some parts of the neointima (Fig. 3E), whereas SM-MHC—a mature SMC marker—was downregulated after ligation injury in the media, as previously reported (Fig. 3F)^24^.

**Figure 4:**
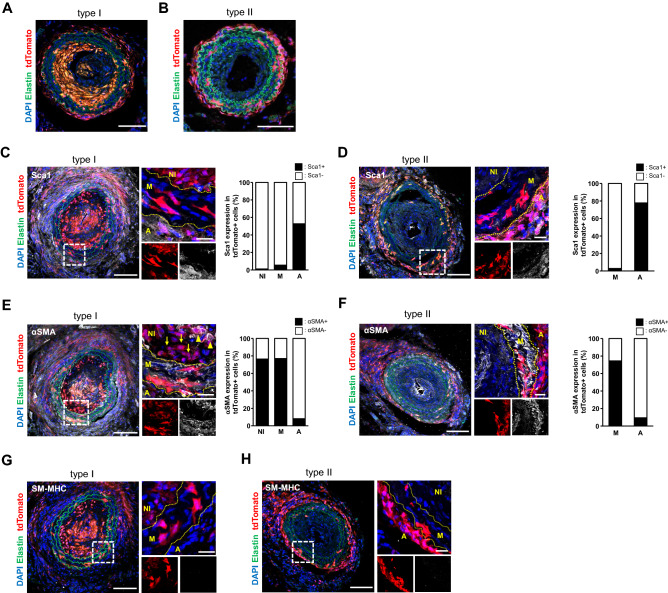
Two types of neointima formed by distinct SMCs after carotid artery ligation. (**A**, **B**) Representative images of type I (**A**) and type II (**B**) neointima at 56 days after ligation. Scale bars: 100 µm. (**C**, **D**) Immunostaining for Sca1 on carotid artery sections from type I (**C**) and type II (**D**) neointima. The dotted boxed area is shown on the right with higher magnification. Quantification of the percentage of Sca1 expressing cells in tdTomato+ cells within the vessel wall. Data are presented as the mean ± SEM, *N* = 3–8 per group. NI: neointima, M: media, A: adventitia. Scale bars: 50 µm. Scale bars in insets: 20 µm. (**E**, **F**) Immunostaining for αSMA on carotid artery sections from type I (**E**) and type II (**F**) neointima. Arrowheads indicate tdTomato+/αSMA+ cells in the neointima. Arrows indicate tdTomato+/αSMA- cells. The dotted box area is shown on the right present an image with higher magnification. Quantification of the percentage of αSMA-expressing cells in tdTomato+ cells within the vessel wall. Data are presented as the mean ± SEM, *N* = 3–8 per group. NI: neointima, M: media, A: adventitia. Scale bars: 100 µm, Scale bars in insets: 20 µm. (**G**, **H**) Immunostaining for SM-MHC on carotid artery sections from type I (**G**) and type II (**H**) neointima. The dotted box area is shown on the right with higher magnification. NI: neointima, M: media, A: adventitia. Scale bars: 100 µm, box, Scale bars in insets: 20 µm.

At 56 days after carotid artery ligation, the neointima was still maintained (Fig. 3G) and a greater abundance of tdTomato+ cells was observed in the neointima (Fig. 3H) (contribution of tdTomato+ cells to neointima: 2 out of 10 mice at 28 days, 8 out of 11 mice at 56 days). The contribution of tdTomato+ cells within the neointima significantly increased at 56 days when compared to observation at 28 days (Fig. 3I). This observation suggests that the neointima is an unstable structure with a dynamic nature and constituents that can change as it matures. Interestingly, we observed two types of neointima at 56 days: in 8 out of 11 mice, the majority of neointimal cells were tdTomato+ (Fig. 4A), while nearly all neointimal cells were tdTomato- in the other 3 mice (Fig. 4B). We defined these two types of neointima as type I (abundant tdTomato+ cells) and type II (few tdTomato+ cells).

To further characterize two types of neointima, we stained the injured vessels with Sca1. In the adventitia, 68% of Sca-1+ cells were tdTomato+ around type I neointima (n = 8), while 78% of Sca-1+ cells were tdTomato+ around type II neointima (n = 3). The overall distribution pattern of Sca1-expressing tdTomato+ cells was similar between the two types (see graphs in Figs. 4C, D). Sca1+ cells were not observed in the neointima or media. On the other hand, approximately 76% of tdTomato+ cells were positive for αSMA in type I neointima (Fig. 4E). Similarly, tdTomato- cells in type II neointima were composed of αSMA+ cells (Fig. 4F), indicating that type II neointimal cells were derived from non-PDGFRa SMCs, although it is still possible that non-recombined PDGFRa+ cells may have contributed to type II neointima. The ratio of αSMA+/tdTomato+ cells in the media and adventitia was comparable between type I and type II neointima (graphs in Figs. 4D, E). Notably, SM-MHC was not observed in any tdTomato+ cells for both types (Figs. 4G, H). Finally, to examine the level of active PDGFRa expression in the neointima, we performed immunostaining using an anti-PDGFRa antibody. Neither type of neointima expressed PDGFRa, whereas tdTomato+ cells in the adventitia expressed PDGFRa in both types (Figures S4D, E). These results suggest that the type I eointima is comprised of PDGFRa progeny, and indicated that PDGFRa+ cells give rise to SMCs that contribute to neointima formation and that these cells are consistent with immature SMCs and distinct from Sca1+ cells.

### PDGFRa+ cells generate SMCs after wire injury

**Figure 5:**
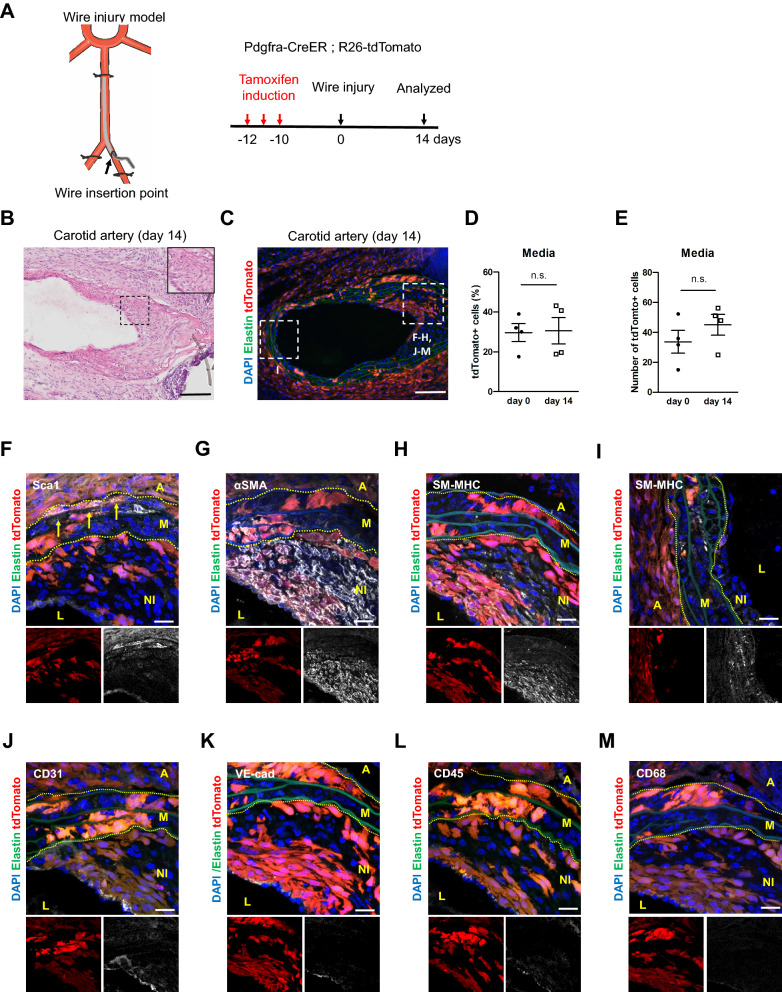
PDGFRa+ cells generate SMCs after wire injury. (**A**) Schematic outlining experimental strategy. (**B**) Hematoxylin eosin-stained carotid artery at 14 days after wire injury. The dotted box area is shown in the inset present an image with higher magnification. Scale bar: 100 µm. (**C**) Fluorescence image showing the distribution of tdTomato+ cells at 14 days after wire injury. Scale bar: 100 µm. (**D**) Quantification of the percentage of tdTomato+ cells in the media before and after wire injury. Data are presented as the mean ± SEM, day 0, *N* = 3, 14 days after wire injury, *N* = 4. Unpaired t-test, n.s.: not significant. (**E**) Quantification of the number of tdTomato+ cells in the media before and after wire injury. Data are presented as the mean ± SEM, day 0, *N* = 3, 14 days after wire injury, *N* = 4. Unpaired t-test, n.s.: not significant. (**F**–**M**) Immunostaining for Sca1 (**F**), αSMA (**G**), SM-MHC (**H**, **I**), CD31 (**J**), VE-cad (**K**), CD45 (**L**), and CD68 (**M**) on carotid artery sections at 14 days after wire injury. Arrows indicate tdTomato+ /Sca1+ cells in the media. L: lumen, I: intima, NI: neointima, M: media, A: adventitia. Scale bars: 20 µm. See also Figure S5.

To determine whether the contribution of the PDGFRa+ cells to neointima formation is context-dependent, we employed a wire injury model. Wire injury is a more severe type of injury performed by inserting a wire into the main carotid artery to remove endothelial cells. We injected tamoxifen into Pdgfra-CreER; R26-tdTomato mice at 2 months of age and performed wire injury after 10 days (Fig. 5A). At 14 days after injury, we observed that tdTomato+ cells contributed in parts of the neointima in all examined mice (n = 4, Figs. 5B, C). The sham-operated control showed no neointima and tdTomato+ cells were preferentially observed in the adventitia (n = 2, Figures S5A, B). Additionally, tdTomato+ cells showed no significant characteristic changes (Figures S5C–H). The ratio and number of tdTomato+ cells in the media remained after wire injury (Figs. 5D, E). Following the immunostaining of tissue sections, it was observed that tdTomato+ cells in the adventitia and outer medial layer were positive for Sca1 (arrows in Fig. 5F); however, the neointima was negative for Sca1 (NI in Fig. 5F). In contrast, neointimal tdTomato+ cells expressed the SMC markers αSMA (Fig. 5G) and SM-MHC (Fig. 5H), suggesting that the PDGFRa-derived cells were mature SMCs or premature SMCs that terminally differentiated within 14 days. SMCs in the media of injured arteries with neointima showed the downregulation of SM-MHC (M in Fig. 5H), whereas SMCs in the vessels free of neointima were SM-MHC+ (M in Fig. 5I). TdTomato+ cells in the neointima were negative for endothelial markers CD31 (Fig. 5J) and VE-cadherin (VE-cad, Fig. 5K) as well as hematopoietic markers CD45 (Fig. 5L) and CD68 (Fig. 5M), excluding the active contribution of endothelial cells or hematopoietic cells. Taken together, the carotid artery rapidly deployed tdTomato+ cells into the neointima and contributed to the accumulation of Sca1- mature SMCs after injury, whereas Sca1+ medial SMCs—which are also marked by PDGFRa+ cells—remained in the medial layers and exhibited differential responses to severe insults, even among PDGFRa+ progeny.

### PDGFRa-derived cells proliferated in response to pressure overload

**Figure 6:**
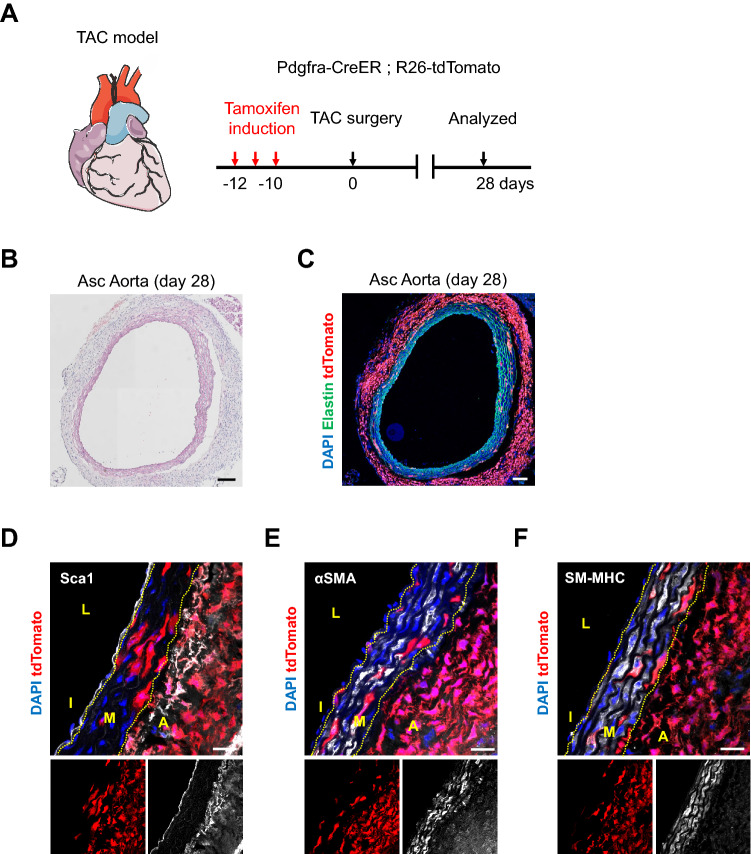
Pressure overload increased PDGFRa-derived cells in the adventitia. (**A**) Schematic outlining the experimental strategy. (**B**) Hematoxylin eosin-stained ascending aorta at 28 days after TAC surgery. Asc Aorta: ascending aorta. Scale bar: 100 µm. (**C**) Fluorescence image showing the distribution of tdTomato+ cells at 28 days after TAC surgery. Asc Aorta: ascending aorta. Scale bar: 100 µm. (**D**–**F**) Immunostaining for Sca1 (**D**), αSMA (**E**), SM-MHC (**F**) on ascending aorta sections at 28 days after TAC surgery. L: lumen, I: intima, M: media, A: adventitia, Asc Aorta: ascending aorta. Scale bars: 20 µm. See also Figure S5.

Finally, we performed transverse aortic constriction (TAC) injury to induce aortic wall thickening via pressure overload (Fig. 6A). At 28 days after TAC, we observed a markedly thickened adventitia of the ascending aorta (Fig. 6B) and an abundance of tdTomato+ cells in the adventitia, indicating adventitial hyperplasia (Fig. 6C). In sham-operated animals, no adventitial hyperplasia was observed and tdTomato+ cells were preferentially located in the adventitia (Figures S5I, J). To further analyze the aortic remodeling with TAC, we performed immunostainings. TdTomato+ cells in the sham-operated control showed no characteristic changes (Figures S5K–M). In contrast, tdTomato+ cells in the adventitia were Sca1+ , and the cells at the boundary between the adventitia and media strongly expressed Sca1 (Fig. 6D). Additionally, tdTomato+ cells in the adventitia were αSMA+ but not SM-MHC+ (Figs. 6E, F), suggesting that these cells are most likely activated fibroblasts derived from PDGFRa+ cells.

## Discussion

**Figure 7:**
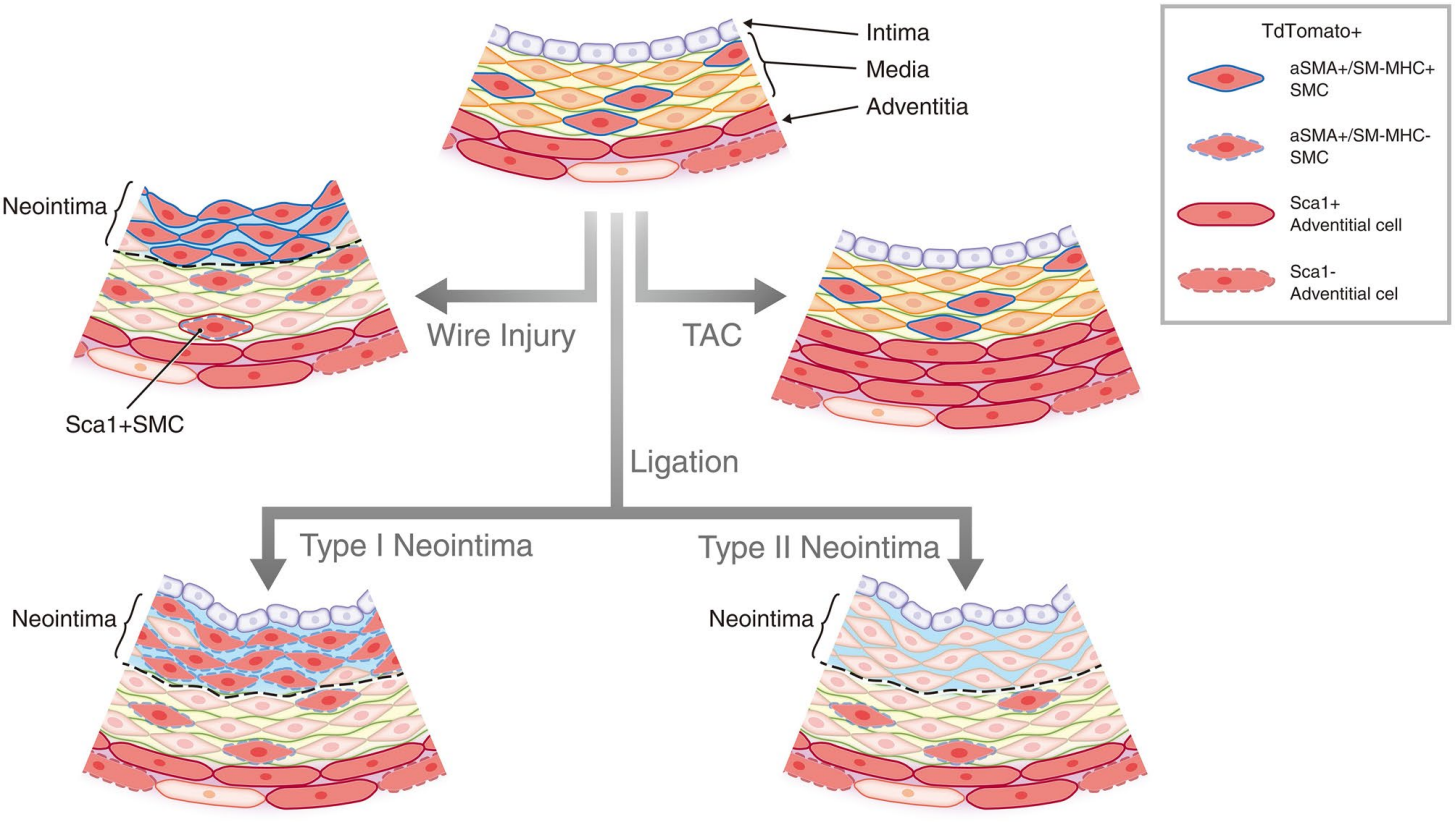
Cellular dynamics of PDGFRa+ cells in response to injuries. Schematic outline of the distinct cellular dynamics of PDGFRa+ cells (red) in response to injuries. The cell populations in the neointima induced by ligation and denudation injuries are heterogeneous and physical insults elicited distinct responses from SMCs that originated from PDGFRa+ cells. In wire injury, adventitial PDGFR+ cells migrated to the media and their daughter cells contributed to neointima formation. Note that tdTomato+ Sca1+ SMC is detected in the medial layer only after wire injury. (CC BY Mayumi Mori).

In this study, we used the combination of a CreER transgenic mouse line that labels PDGFRa+ derived cells predominantly in the adventitia and subsets of SMCs, and three injury models to elucidate the cellular dynamics of PDGFRa+ cells in homeostasis, neointima formation, and vascular remodeling. We observed that a significant number of PDGFRa+ cells in the adventitia were positive for stem cell markers, Sca1, and CD34. We also observed that cell populations in the neointima induced by ligation and denudation injuries were heterogeneous and physical insults elicited distinct responses from SMCs that originated from PDGFRa+ cells (Fig. 7).

To date, the vascular progenitor cells described in the literature include smooth muscle progenitor cells (Sca1+/CD34+ or CD34-), multipotent vascular stem cells, mesenchymal stem/stromal cells (CD29+/CD44+/CD105+/CD90+/CD45-/CD34+ or CD34-/Sca1+ or Sca1-/CD31-/Nestin+ /Gli +), and endothelial progenitor cells (CD34+ /CD31+ /Sca1+ /Flk+ /cKit+ /CD45-)^25^. Multipotent vascular stem cells or mesangioblasts are important in postembryonic mesoderm development, originate from the dorsal aorta, and are characterized by high VEGFR2/Flk1 expression, which makes them an important source of vascular stem cells^25^. Previous studies have assessed the roles of bone marrow and circulating endothelial progenitor cells in new vessel formation. While these roles remain open for debate, an increasing number of studies are looking towards the vascular wall as a stem/progenitor niche^26^. Our study demonstrates that PDGFRa+ cells give rise to long-lived SMCs (up to 2 years) in the vessel wall.

Among these candidate resident vascular stem/progenitor cells, the adventitial layer has been singled out as an active niche for several types of stem/progenitor cells, specifically within a small region adjacent to the medial layer known as the “vasculogenic zone”^27,28^. A population of cells with progenitor markers in the adventitia (Sca1+ , cKit+ , and CD34 +) were also documented in atherosclerosis-prone apolipoprotein E homozygous knockout mice, and it was noted that these were likely progenitor cells native to the vessel wall^13^. Recently, it has been shown that Sca1-CreER+ tdTomato-labeled cells, which are also positive for PDGFRa, do not generate SMCs during homeostasis or contribute to neointima formation after wire injury. However, these can generate SMCs that contribute to vascular remodeling after anastomosis, which is a more severe form of damage when compared to wire denudation^18^. These Sca1+ cells are present in the adventitia before injury and can also be found in the medial layer after anastomosis, expressing typical SMC markers such as SMA, SM22, CNN1, and SM-MHC. SMC turnover is suggested as the main contributor to neointima formation after wire injury, while adventitial cells are only capable of SMC differentiation after severe anastomosis injury^22^. In the present study, we observed two populations of PDGFRa-derived cells inside the ligation-induced neointima, which were characterized by αSMA+ /tdTomato+ as well as αSMA-/tdTomato+ , both of which are negative for Sca1 (Type I, Fig. 4E). At 28 days post-ligation injury, there was an overall lower contribution of PDGFRa+ cells to neointima formation. However, this contribution increased after 56 days, suggesting that neointima maintenance occurs with an exchange of SMCs in a temporally regulated manner.

In wire injury, we even observed a clear contribution of tdTomato+ cells in the neointima at 14 days after injury. Ki67 staining showed low proliferation in neointima, and Ki67-positive cells were mostly negative for tdTomato (data not shown). Interestingly, wire injury showed SM-MHC+ cells in both the medial layer and neointima, indicating that PDGFRa+ medial SMCs most likely migrated to the neointima after wire denudation. Two distinct injury models demonstrated that the neointima composition is different in ligation vs denudation. Possible reasons for the temporal contribution of PDGFRa+ cells and differential response to injury could include the endothelial-dependent pathways between endothelial cells and SMCs. During vascular homeostasis, endothelial cells and SMCs maintain a balance of vasodilation and vasoconstrictor factors. A disrupted intima layer and stress caused by prolonged blood flow alteration may have caused endothelial dysfunction, which likely influences the migration of SMCs into the neointima via the increased presence of SMC activators^29^.

The present study highlights a prominent proliferative response of PDGFRa+ cells, which appeared in adventitial fibroblasts after pressure overload and exhibited aortic fibrosis. PDGFRa+ adventitial cells can sense mechanical stress and induce vessel wall thickening via the over-proliferation of fibroblasts and synthesis of a large amount of extracellular matrix to protect the aortic wall, which is consistent with previous reports^30^. Adventitial fibroblasts have also been shown to promote neointima formation by inducing PDGF-mediated sonic hedgehog (SHH) signaling via a non-canonical pathway (Gli-independent) and producing cytokines such as CXCL1, IL-6, and IL-8^31^. Therefore, adventitial cells can indirectly affect neointima formation.

Notably, we observed a positive relationship between the severity of vascular injuries and mobilization of PDGFRa+ cells into the neointima. Whereas the response of PDGFRa+ cells to the ligation-induced neointima is a slow process, wire denudation rapidly mobilized PDGFRa+ cells into the neointima and upregulated SMC markers within 14 days. Additionally, our data indicate that PDGFRa+ /Sca1+ cells migrate to the media and their daughter cells contribute to neointima formation. Although we could not decipher whether adventitial PDGFRa+ cells migrated through medial layers to form neointima or PDGFRa+ SMCs in the medial layer responded to form neointima, our data support a crucial role of PDGFRa+ cells in the acute and severe injury response. A recent report showing that Sca1+ cells do not contribute to homeostasis or wire injury response^18^ suggests that PDGFRa+ and Sca1+ cells are distinct in their threshold and capacity to respond to injury and that the progeny of PDGFRa+ and Sca1+ adventitial cells give rise to a distinct subpopulation of neointima cells. It is also possible that this difference may be due to differential microenvironment between the carotid and femoral arteries. Notably, FACS analysis would be useful to further characterize distinct populations in the neointima: αSMA+ /tdTomato+ and αSMA-/tdTomato+ cells. Our study underscores the heterogeneity of adventitial cells and their differential responses and mobilization upon injury. The further molecular characterization of cells derived from PDGFRa+ , Sca1+ or Gli1+ will be crucial to understand the dynamics of vascular cells during homeostasis and injury responses.

## Methods

### Mice

B6.Cg-Pdgfra < tm1.1(EGFP/cre/ERT2) Hyma > (hereafter Pdgfra-CreER) mice were generated in the laboratory of Dr. Takumi Era^20^ and provided by the RIKEN BRC through the National Bio-Resource Project of the MEXT, Japan. Rosa26-*loxP-stop-loxP*-tdTomato (hereafter R26-tdTomato) mice were purchased from Jackson Laboratory (JAX007905). All experimental mice were kept on a 12 h/12 h light/dark cycle under specific pathogen free conditions in the Laboratory Animal Resource Center at the University of Tsukuba prior to the experiments. For lineage tracing studies, 2-month-old mice received 3 × 100 µg/g bodyweight tamoxifen (Sigma T5648) in corn oil (Sigma C8267) via intraperitoneal injection 10 days before surgery, unless otherwise noted. For the 2-year-chase, we used a 5 × 100 µg/g bodyweight tamoxifen protocol. All animal procedures were conducted following animal experimentation guidelines approved by the Institutional Animal Experiment Committee at the University of Tsukuba (APN# 20-262) and the Animal Research: Reporting of In Vivo Experiments (ARRIVE) guidelines.

### Injury models

Carotid artery ligation was performed as previously described^32^. Briefly, male mice were anesthetized by an intraperitoneal injection of tribromoethanol (250 mg/Kg) and the left carotid artery was exposed through a surface neck incision and ligated twice with a black silk suture. Mice were sacrificed at 28 and 56 days after surgery, and the right and left carotid arteries were harvested.

Wire injury of the carotid artery was conducted using a previously described method with modification^33^. The left carotid was exposed similarly to ligation. The carotid was clamped with silk at both bifurcation points (internal and external carotid arteries) and the main carotid point to restrict blood flow from the heart, after which a small incision was made at the external carotid artery (ECA) bifurcation. After draining the blood, a 0.38 mm/0.015 inch fixed core wire guide (#C-SF-15-15, COOK Inc) was inserted in the incision and moved back and forth 10 times into the main carotid. After wire insertion, the main carotid clamp was released to allow the blood flow to flush out the vascular wall cell debris and the incision at the ECA was closed by ligating the ECA incision after the bifurcation point. After checking the incision point for leakage, blood flow was restored to the main carotid and the skin was sutured to complete the surgery. Tissues were collected at 14 days after wire injury surgery and serial sections were prepared. In both carotid artery injuries, sham-operated mice served as controls.

Mice underwent transverse aortic constriction (TAC) using a standard surgical protocol^34^. Briefly, anesthetized mice were placed in a supine position, and TAC was achieved by tying a 6–0 silk suture (Natsume Seisakusho Co., Ltd.) against a 25-gauge blunt needle (TERUMO). For the sham group, the same operation was performed without ligating the aorta. At 28 days after TAC, mice were sacrificed and the aortas were harvested. Transverse sections were then prepared from at least three different levels proximal to the constriction.

### Immunohistochemistry

Carotid arteries and aortas were harvested and prefixed in 4% paraformaldehyde for 1 h at room temperature, and incubated in 30% sucrose for cryopreservation. Tissues were embedded in optimal cutting temperature (OCT) compound (Tissue-Tek; Sakura Finetek) and cryosectioned (10 μm) using a cryostat (NX70, Thermo Fisher Scientific). Sections were stained with hematoxylin and eosin for routine histology. For immunohistochemistry, sections were permeabilized in phosphate buffered saline (PBS) containing 0.1% Triton, and blocked in PBS with 5% normal serum. Then, sections were stained with mouse anti-αSMA (1A4) (Sigma Aldrich), rabbit anti-SM-MHC (Biomedical Technologies/ Alpha Aesar BT-562), goat PDGFRa (R&D Systems AF1062), rat anti-CD31 (MEC 13.3), rat anti-CD34 (RAM34), rat anti- CD45 (30-F11), rat anti-Sca1 (E13-161.7) (BD Biosciences), mouse anti-VE-cadherin (F-8), and mouse anti-CD68 (KP1) (Santa Cruz Biotechnology) used at 1:100. Secondary reagents were Alexa Fluor 647 conjugated antibodies from Invitrogen used at 1:200. Slides were covered with Vectashield containing DAPI (Vector Laboratories) and viewed under an LSM 710 laser scanning microscope (Zeiss) or Axio Imager.Z2 (Zeiss). Images were analyzed for quantification using Fiji Image J. At least three mice were used for each experiment (except *N* = 2 in Fig. 2 and Figure S4), and at least 10 sections were examined.

### Statistical analysis

All values are represented as mean ± SEM. A Shapiro–Wilk test was used for the normality test. When the data followed normal distribution, statistical significance was determined by either unpaired Student t-test, and when the assumption of Normality was violated, Mann–Whitney test was performed (Prism 8, Graph Pad, ver. 8.4.0). *P* < 0.05 denotes statistical significance.

## Supplementary Information

Supplementary Information
