# Supplementary material for: Contribution of PDGFRα-positive cells in maintenance and injury responses in mouse large vessels
Source: Sci Rep. 2021 Apr 21;11:8683. doi: 10.1038/s41598-021-88126-6 (PMC8060414; doi:10.1038/s41598-021-88126-6)
Supplement: Supplementary file 1 — Supplementary Information [file 41598_2021_88126_MOESM1_ESM.pdf]

Contribution of PDGFRα-positive cells in maintenance and injury responses in mouse large vessels

Kenichi Kimura[1†], Karina Ramirez[1,2†], Tram Anh Vu Nguyen [1,2], Yoshito Yamashiro[1], Aiko Sada[1,3] and Hiromi Yanagisawa[1,4]

[1] Life Science Center for Survival Dynamics, Tsukuba Advanced Research Alliance (TARA), University of Tsukuba, Tsukuba, Japan.

[2] Ph.D. Program in Human Biology, School of Integrative and Global Majors, University of Tsukuba, Tsukuba, Japan.

[3] International Research Center for Medical Sciences (IRCMS), Kumamoto University, Kumamoto, Japan

[4] Faculty of Medicine, University of Tsukuba, Tsukuba, Japan.

† These authors contributed equally.

Correspondence:

Hiromi Yanagisawa, M.D., Ph.D.
Life Science Center for Survival Dynamics, Tsukuba Advanced Research Alliance,
University of Tsukuba,
1-1-1 Tennodai, Tsukuba, Ibaraki 305-8577, Japan
Tel: +81-29-853-7318
Fax: +81-29-853-7322
Email: hkyanagisawa@tara.tsukuba.ac.jp

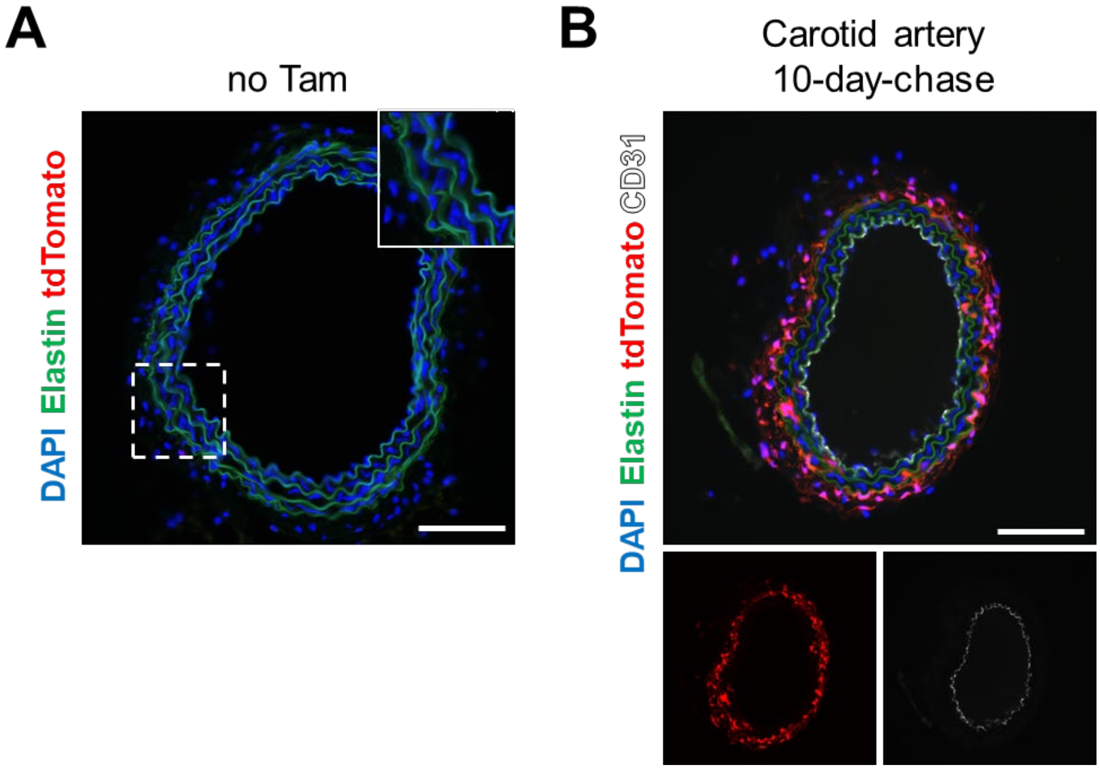

Supplementary Figure S1

Representative images of PDGFRa+ cells in aortas (related to Figure 1).

(A) Labeling after the no-tamoxifen condition at 2 months of age. The dotted box area is shown in the inset presents an image with higher magnification. Scale bar: 100 μm.

(B) Immunostaining for CD31 on carotid artery sections from 10-day-chase. Scale bar: 100 μm.

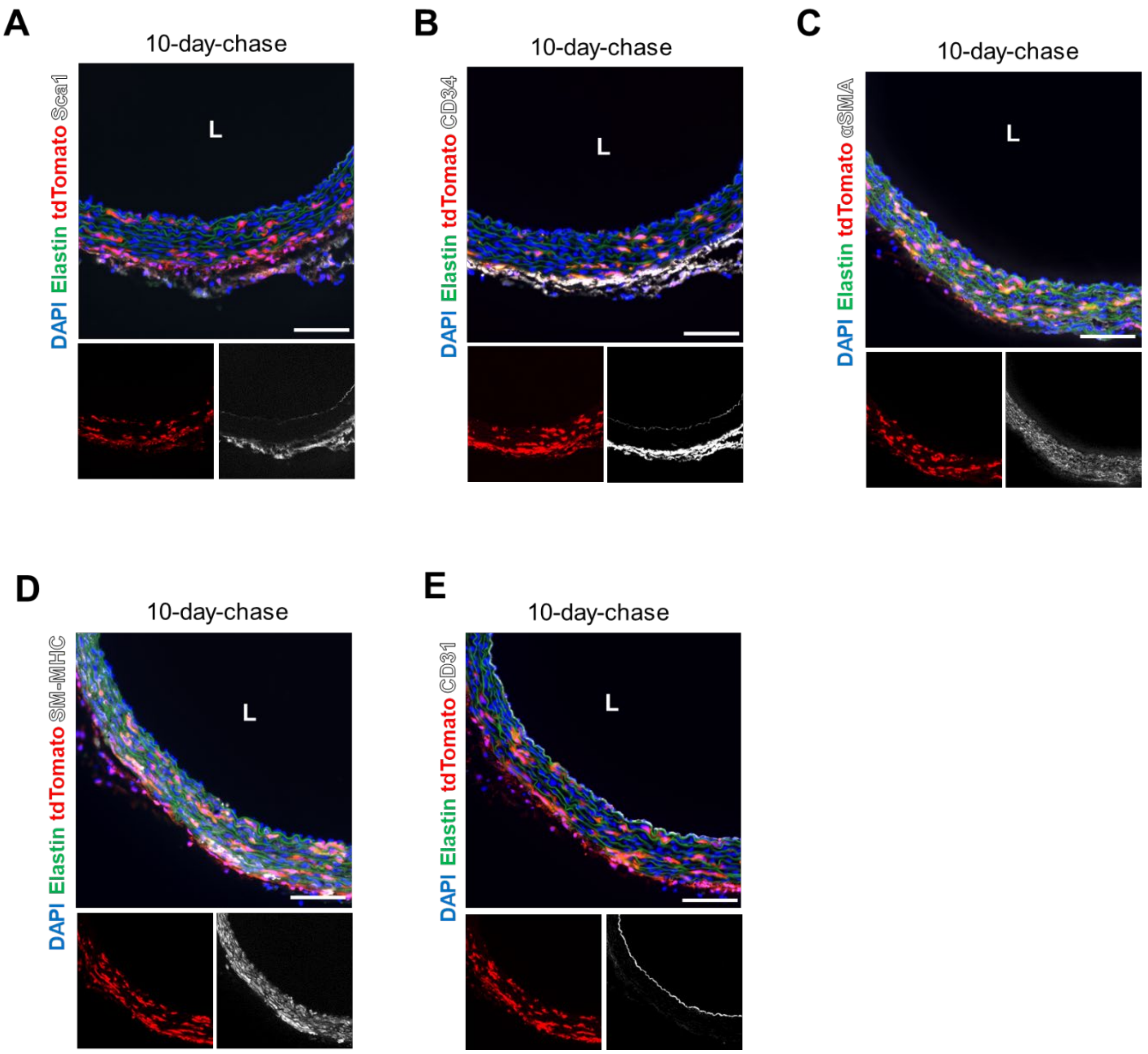

Supplementary Figure S2

Genetic labeling of PDGFRa+ cells in the ascending aorta (related to Figure 1).

(A-E) Immunostaining for Sca1 (A), CD34 (B), αSMA (C), SM-MHC (D), and CD31 (E) on ascending aorta sections from 10-day-chase. L: lumen. Asc Aorta: ascending aorta. Scale bars: 100 μm.

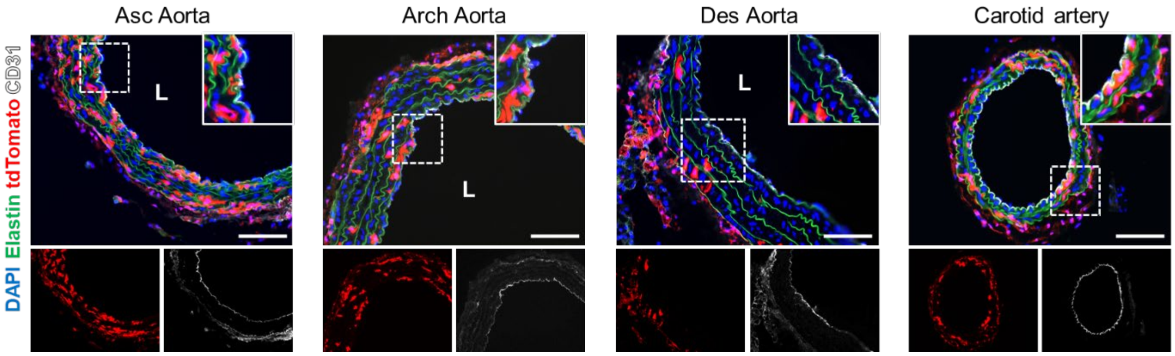

Supplementary Figure S3

Characterization of PDGFRa+ cells during a long-term homeostasis (related to Figure 2).

Comparison of CD31 immunostaining among different portions of large arteries (Asc Aorta, Arch of Aorta, Des Aorta, Carotid artery) at 2-year-chase. The dotted box area shown in the inset represents an image with higher magnification. L: lumen. Scale bars: 100 μm.

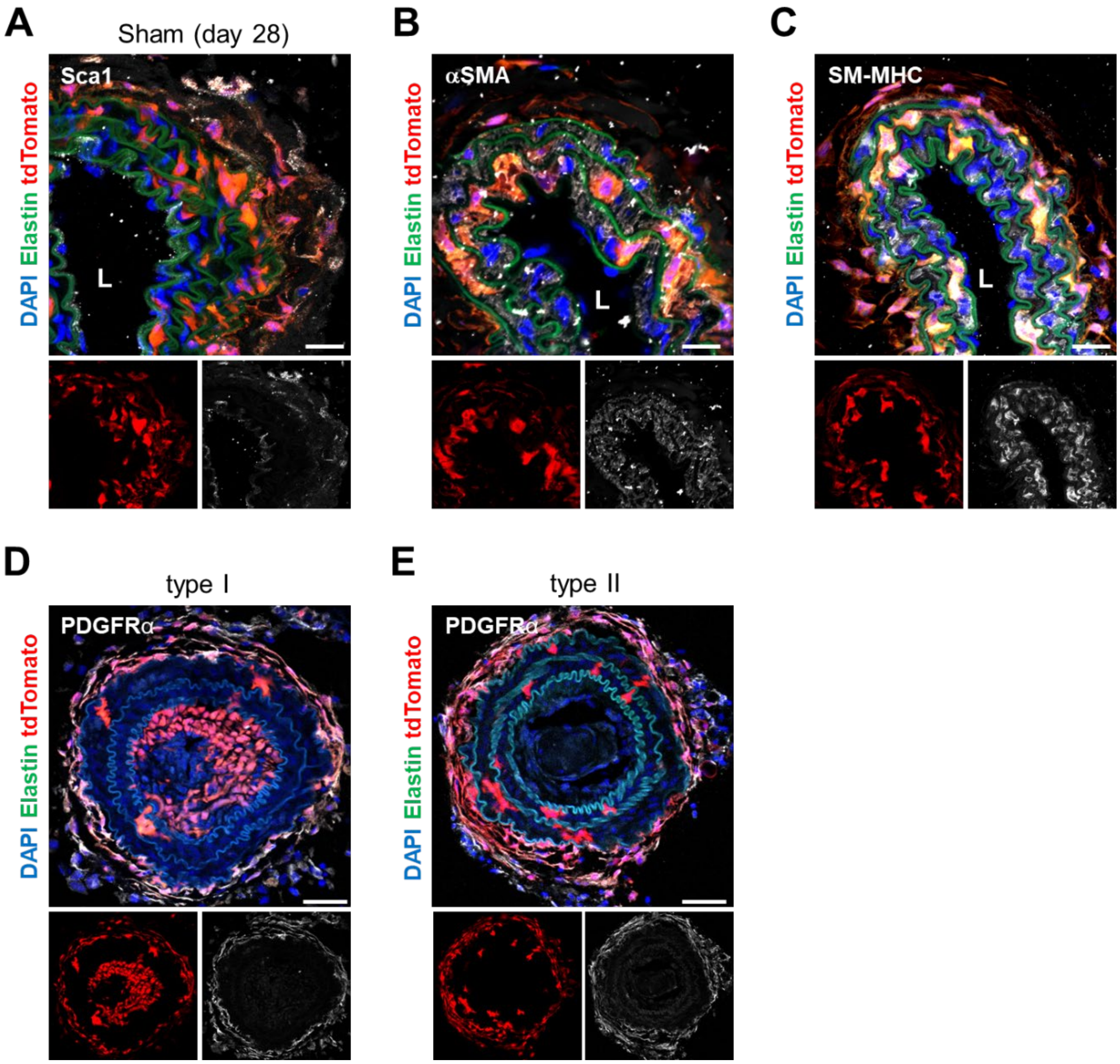

Supplementary Figure S4

Characterization of PDGFRa+ cells in the sham-operated carotid artery (Related to Figures 3 and 4).

(A-C) Immunostaining for Sca1 (A), αSMA (B), and SM-MHC (C) on carotid artery sections at 28 days after the sham operation. L: lumen. Scale bars: 20 μm.

(D and E) Immunostaining for PDGFRα on carotid artery sections from type I (D) and type II (E) neointima. Scale bars: 50 μm.

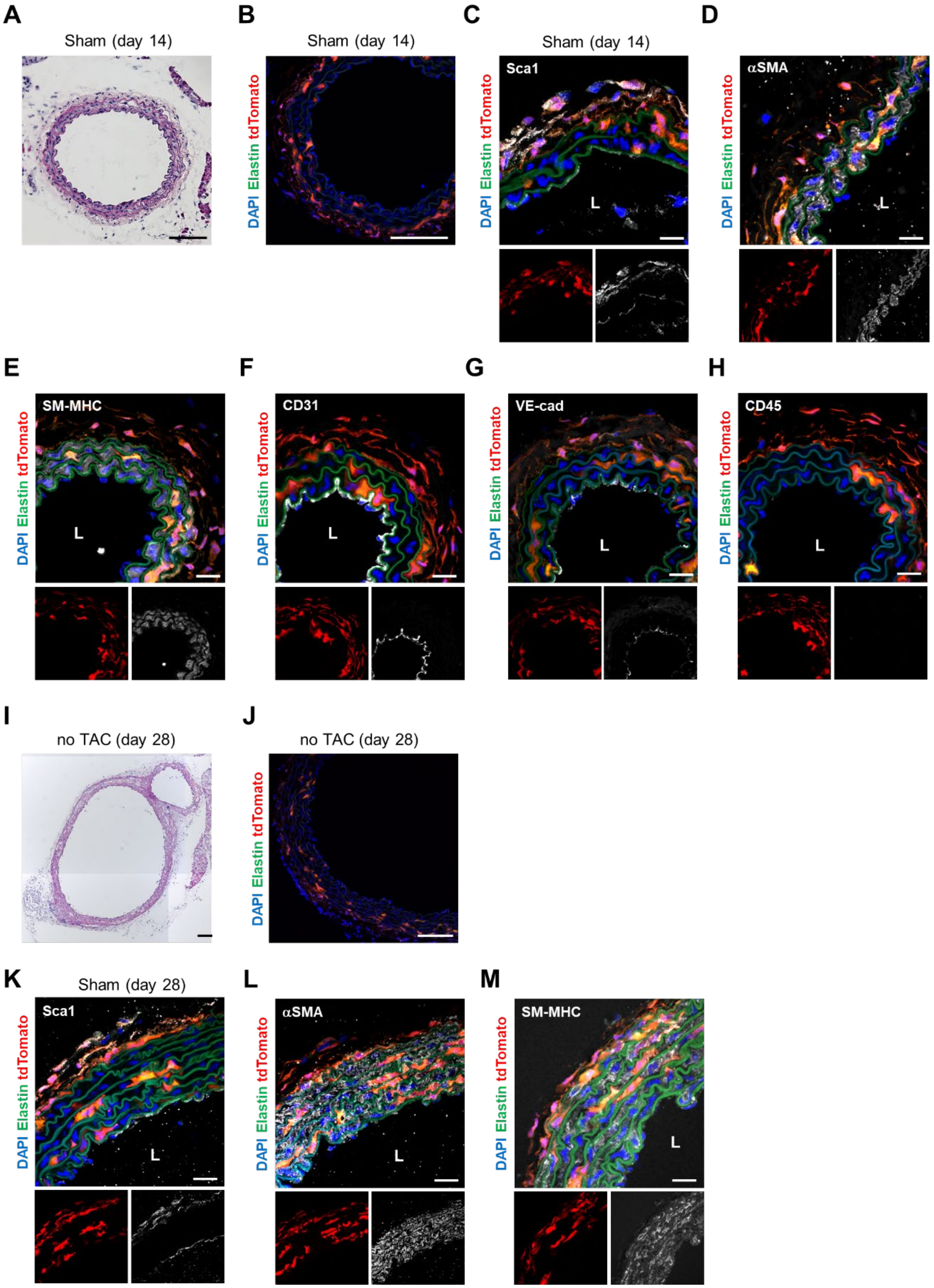

A
Sham (day 14)
B
Sham (day 14)
DAPI Elastin tdTomato
C
Sham (day 14)
Sca1
DAPI Elastin tdTomato
L
D
αSMA
DAPI Elastin tdTomato
L
E
SM-MHC
DAPI Elastin tdTomato
L
F
CD31
DAPI Elastin tdTomato
L
G
VE-cad
DAPI Elastin tdTomato
L
H
CD45
DAPI Elastin tdTomato
L
I
no TAC (day 28)
J
no TAC (day 28)
DAPI Elastin tdTomato
K
Sham (day 28)
Sca1
DAPI Elastin tdTomato
L
L
αSMA
DAPI Elastin tdTomato
L
M
SM-MHC
DAPI Elastin tdTomato
L

Supplementary Figure S5

Distribution of PDGFRa+ cells in aortas after the sham operation (related to Figures 5 and 6).

(A) Hematoxylin eosin-stained carotid artery at 14 days after the sham operation. Scale bar: 100 μm.

(B) Fluorescence image showing the distribution of tdTomato+ cells at 14 days after the sham operation. Scale bar: 100 μm.

(C-H) Immunostaining for Sca1 (C), αSMA (D), SM-MHC (E), CD31 (F), VE-cad (G), and CD45 (H) on carotid artery sections at day 14 after sham operation. L: lumen. Scale bars: 20 μm.

(I) Hematoxylin eosin stained ascending aorta at 28 days after the sham operation. Scale bar: 100 μm.

(J) Fluorescence image showing the distribution of tdTomato+ cells at 28 days after the sham operation. Scale bar: 100 μm.

(K-M) Immunostaining for Sca1 (K), αSMA (L), and SM-MHC (M) on ascending aorta sections on day 28 after the sham operation. L: lumen. Scale bars: 20 μm.
